# Red Wine Extract Prevents Oxidative Stress and Inflammation in ARPE-19 Retinal Cells

**DOI:** 10.3390/cells12101408

**Published:** 2023-05-17

**Authors:** Clarisse Cornebise, Maude Perus, François Hermetet, Josep Valls-Fonayet, Tristan Richard, Virginie Aires, Dominique Delmas

**Affiliations:** 1 UFR des Sciences de Santé, Université de Bourgogne, 21000 Dijon, France; clarisse.cornebise@gmail.com (C.C.); maude.perus@u-bourgogne.fr (M.P.); francois.hermetet@u-bourgogne.fr (F.H.); virginie.aires02@u-bourgogne.fr (V.A.); 2 INSERM Research Center U1231—Cancer and Adaptive Immune Response Team, Bioactive Molecules and Health Research Group, 21000 Dijon, France; 3 Université de Bordeaux, Bordeaux Sciences Agro, Bordeaux INP, INRAE, OENO, UMR 1366, ISVV, 33140 Villenave d’Ornon, France; josep.valls-fonayet@u-bordeaux.fr (J.V.-F.); tristan.richard@u-bordeaux.fr (T.R.); 4 Centre de Lutte Contre le Cancer Georges François Leclerc Center, 21000 Dijon, France

**Keywords:** polyphenol, red wine extract, resveratrol, AMD, oxidative stress, inflammation, prevention

## Abstract

Age-related macular degeneration (AMD) is one of the most commonly occurring ocular diseases worldwide. This degenerative condition affects the retina and leads to the loss of central vision. The current treatments are focused on the late stage of the disease, but recent studies have highlighted the importance and benefits of preventive treatments and how good dietary habits can reduce the risk of progression to an advanced form of the disease. In this context, we studied whether resveratrol (RSV) or a polyphenolic cocktail, red wine extract (RWE), are able to prevent the initiating events of AMD (i.e., oxidative stress and inflammation) in human ARPE-19 retinal pigment epithelial (RPE) cells and macrophages. This study highlights that RWE and RSV can prevent hydrogen peroxide (H_2_O_2_) or 2,2′-Azobis(2-methylpropionamidine) dihydrochloride (AAPH)-induced oxidative stress and can subsequently prevent DNA damage via the inhibition of the ATM (ataxia telangiectasia-mutated)/Chk2 (checkpoint kinase 2) or Chk1 signaling pathways, respectively. Moreover, ELISA assays show that RWE and RSV can prevent the secretion of proinflammatory cytokines in RPE cells and in human macrophages. Interestingly, RWE exhibits a greater protective impact compared to RSV alone, even though RSV was more concentrated when used alone than in the red wine extract. Our results suggest that RWE and RSV may have potential interest as preventive nutritional supplementations against AMD.

## 1. Introduction

Age-related macular degeneration (AMD) is currently the leading cause of visual impairment in people over the age of 50 in developed countries, affecting hundreds of millions of individuals worldwide [1]. The risk of AMD occurrence increases with age to exceed 25% of the population after the age of 75 [2]. This disease mainly affects the macula, which is localized in the center of the retina and surrounds the fovea, responsible for the high quality and color central vision. AMD can therefore cause blurred vision in the center of the visual field but hardly affects the peripheral vision. Patients with AMD develop one or more of the following retinal abnormalities: drusen (whitish/yellowish deposits of lipid and lipoprotein material between the retinal pigment epithelium (RPE) and Bruch’s membrane, variable in size, shape, and number); alteration or geographic atrophy (GA) of the RPE, or choroidal neovascularization (CNV). These abnormalities depend on the stage of the disease and can be unilateral (only one eye is affected) or bilateral (both eyes are affected). The term AMD corresponds to the most advanced stages of the disease. There are generally three major clinical forms [3,4]. The first is age-related maculopathy (AML), corresponding to the early stage of the disease (appearing around 50 years of age) and characterized by the accumulation of precursors such as drusen of variable shape, size, and number, an accumulation of photoreceptor phagocytosis residues by RPE cells (slowing down of the elimination capacities due to the aging of the retina) that seems to be the origin of the disease. The second is atrophic or “dry” AMD, which is one of the two severe forms of AML, evolving very slowly over time and characterized by a progressive loss of RPE cells due to their degeneration by apoptosis. These events cause the disappearance of the choriocapillaris and photoreceptors in the macular region, leading to severe vision loss. The third is neovascular AMD (nAMD, also called wet or exudative AMD), characterized by neovascularization of the choroid, which accounts for 90% of severe vision loss caused by AMD [5]. The new blood vessels tend to be very fragile and lead to a diffusion of the serum and blood, causing hemorrhages within the retina that disrupt its organization, particularly due to the appearance of exudates, intraretinal edema, scars, or retinal detachment. Since the 2000s, nAMD has been treated by repeated injections of antibodies directed against the major factor regulating angiogenesis, vascular endothelium growth factor A (VEGF-A), directly into the eye via the intravitreal route (on average, seven shots per year). Over the last few years, these targeted therapies have supplanted thermal laser photocoagulation techniques or photodynamic therapies used to destroy new vessels or by creating direct damage to abnormal vessels while preserving healthy retinal tissue [6,7,8]. However, persistent fluid or recurrent exudation may persist despite anti-VEGF therapy, and patients can develop mechanisms of resistance to anti-VEGF treatments in the long term, which consequently reduces the therapeutic efficacy [9,10]. The numerous molecular mechanisms underlying AMD are complex and still not fully understood. However, there are three major elements that lead to the pathogenesis of AMD: major oxidative stress linked to aging, which can induce DNA damage in RPE cells and cause their death, which appears be the triggering factor for AMD [11,12,13,14]; chronic inflammation, which is, in part, characterized by the recruitment and infiltration of proinflammatory immune cells such as macrophages; neoangiogenesis, caused by proangiogenic cytokines; and chemokines, produced by proinflammatory immune cells, which a common feature of the most advanced and severe stages of the disease [15,16]. Many data from the literature also suggest that preventive treatments and/or good eating habits can reduce the risk of progression to an advanced form of the disease: regular consumption of fruits; green vegetables rich in lutein (spinach, broccoli, cabbage curly fish, etc.); fatty fish (salmon, tuna, mackerel, etc.); regular physical activity; etc. Indeed, the controlled, randomized Age-Related Eye Disease Study 1 and 2 (AREDS-1 and AREDS-2) clinical studies demonstrated that nutritional supplementation with antioxidant vitamins such as vitamins C or E, zinc, polyunsaturated fatty acids from the omega-3 family, or lutein can significantly reduce the risk of developing severe forms of the disease [17]. Among antioxidants, resveratrol (RSV), a grapevine polyphenol, could be of major interest due to its pleiotropic properties in oxidative stress, inflammation, and degeneration [18,19,20,21]. Recently, we highlighted the use of this hydroxystilbene as a potential preventive tool against various ocular diseases—in particular, AMD, glaucoma, cataract diabetic retinopathy, and vitreoretinopathy [22]. Through its antioxidant and anti-inflammatory properties, RSV could protect against several deleterious environmental factors that initiate AMD [23], such as air pollution; smoking; ultraviolet radiation; metabolic diseases (e.g., diabetes, hypertension, and obesity); dietary fat consumption [24,25,26,27]; and genetic polymorphisms such as *cfh* and *arms2/hrta1* genes [28]. Alone or combined, these factors could contribute to the initiation of AMD through the production of free radicals such as superoxide anion (O_2_^·−^), nitric oxide (NO^·^), or hydroxyl radical (OH^·^), which generate oxidative stress and inflammation in ocular tissues. More recently, we showed that a mixture of polyphenols, rather than RSV alone, could be more efficient in counteracting some aspects of AMD pathogenesis. We demonstrated that a dry extract of red wine (RWE), containing low amounts of RSV, was able to reduce the secretion of VEGF-A by the human RPE cell line ARPE-19 to a greater extent than 20 µM RSV alone [27]. This effect could be explained by synergic interactions between the compounds present in the mixture, as we previously observed for RSV and the flavonoid quercetin in a colorectal cancer context [29]. Mechanistically, the reduction of the secreted VEGF-A levels resulted from the action of the polyphenol mixture on the VEGF receptor (VEGF-R)/mitogen-activated protein kinases (MAPKs) molecular pathway [30]. Hence, the use of such a formulation may be of value for an AMD prevention strategy.

To better characterize the potential of RWE in AMD, we sought to determine the impact of RWE and RSV on oxidative stress and inflammation, two well-recognized primary triggers of AMD [31]. Herein, we show that RWE was able to significantly decrease the secretion of VEGF-A induced by the recombinant human VEGF-A (rVEGF) protein but, also, by oxidative stress. This inhibition of VEGF-A secretion is associated with a significant reduction in reactive oxygen species (ROS) induced by oxidative agents. Moreover, the decrease of total ROS by RWE is associated with a disruption in the DNA damage pathway. Very interestingly, we demonstrate for the first time that RWE prevents inflammation, a second key initiator of AMD, by reducing the secretion of interleukin (IL)-6 and IL-8 in both RPE cells and immune cells such as macrophages. These findings provide new evidence that a polyphenol combination could be a robust and promising tool to prevent the development of AMD in at-risk populations and to develop innovative prevention strategies.

## 2. Materials and Methods

### 2.1. Red Wine Extract (RWE) Extraction, Purification, and Characterization

Red wine extract (RWE) was obtained from red wine, a Santenay 1^er^ Cru Les gravières 2012 (EARL Capuano-Ferrari Santenay, Côte-d’or, France) that was selected by the Bureau Interprofessionnel des Vins de Bourgognes (BIVB, Beaune, France) and provided by the Centre Technique Interprofessionnel de la Vigne et du Vin (CTIVV, Beaune, France). A dry red wine powder was extracted and analyzed as previously described [30,32,33]. Briefly, alcohol and water were evaporated using a rotary evaporator; then, phenolic compounds were purified using an absorbance column (Diaion^®^ HP-20, 13606, Merck, Darmstadt, Germany) and eluted in alcohol. After elution, a rotary evaporator was used to evaporate all the alcoholic eluent, and the extract was then concentrated to dryness.

The composition of RWE was characterized as previously described [30]. Briefly, Thermo Scientific Vanquich UHPLC equipped with a Thermo Scientific MWL detector was used to analyze the anthocyanins. The other polyphenols were analyzed by high-performance liquid chromatography coupled to an Agilent 6430 triple-quadrupole mass spectrometer (HPLC-MS/MS), as previously published [30]. In brief, compounds were separated with an Agilent 1260 HPLC and eluted on an Agilent Zorbax SB-C18 column. Specific Multiple Reaction Monitoring (MRM) transitions and retention times were used for the detection and quantification of each compound. All compounds were quantified as their corresponding standard except flavan 3-ol dimers B3 and B4, which were expressed as dimers B1 and B2, respectively, and cis-resveratrol and cis-piceid, which were quantified as their respective trans-isomer forms [30].

### 2.2. Chemical Reagents and Antibodies

Trans-resveratrol (RSV, R5010, Sigma-Aldrich, St. Quentin Fallavier, France) stock solution was prepared at 20 mM in 70% ethanol and stored at −20 °C. A stock solution of lipopolysaccharides from Escherichia coli O55:B5 (LPS, L2880, Sigma-Aldrich, St. Quentin Fallavier, France) was prepared in H_2_O at 5 mg/mL and stored at −20 °C. Human recombinant interferon gamma (IFN-γ, 300-02, preprotech, Neuilly-Sur-Seine, France) stock solution was prepared in H_2_O at 25 µg/mL and stored at −20 °C. Recombinant human VEGF-165 (rVEGF) protein (11858821, Invitrogen, Waltham, MA, USA) was solubilized in H_2_O at 50 µg/mL and stored at −20 °C. 2′,7′-Dichlorofluorescin diacetate (DCFH-DA, D6863, Sigma-Aldrich, St. Quentin Fallavier, France) was prepared in dimethyl sulfoxide (DMSO); the stock solution was prepared at 50 mM and stored at −20 °C. Hydrogen peroxide (H_2_O_2_) was purchased from MERCK (H1009, Darmstadt, Germany), and a stock solution was extemporaneously prepared at 300 mM in culture medium for cell treatments. 2,2′-Azobis(2-methylpropionamidine) dihydrochloride (AAPH; 440914, MERCK) was prepared as a stock solution at 600 mM in water and was stored at −20 °C.

For the Western blot analyses, the following antibodies were used: ATM (#2873, 1:1000); *p*-ATR (#2853, 1:1000); Chk1 (#2360, 1:1000); *p*-Chk1 (#2348, 1:1000); Chk2 (#2662, 1:1000); *p*-Chk2 (#2197, 1:1000); p53 (#2524, 1:1000); and *p*-p53 (#9284, 1:1000), purchased from Cell Signaling Technology (Ozyme, Saint-Cyr-l’Ecole, France). ATR (Sc-1887, 1:200); p21 (Sc756, 1:500); and heat shock cognate (HSC70) (sc-7298; 1:10,000) were purchased from Santa Cruz Biotechnology (Nanterre, France). *p*-ATM (ab81292, 1:50,000) was purchased from Abcam (Cambridge, UK). For the immunofluorescence analyses, *p*-Histone H2A.X (#80312, 1:200) was purchased from Cell Signaling Technology (Ozyme, Saint-Cyr-l’Ecole, France), and anti-mouse 488 fluorophore-conjugated secondary antibody (SAB4600387, 1:500) was obtained from Sigma-Aldrich (St. Louis, MO, USA).

For flow cytometry, Alexa Fluor^®^ 647 anti-*p*-histone H2A.X (Ser139) antibody was purchased from BioLegend (San Diego, CA, USA).

### 2.3. Cell Culture and Treatments

#### 2.3.1. Human Retinal Cell Line ARPE-19

The human RPE cells (ARPE-19, CRL-2302^TM^) were purchased and validated by the American-type Cell Culture Collection (ATCC) (Manassas, MD, USA). Nonpolarized cells were cultured in Dulbecco’s modified Eagle’s medium; the nutrient mixture F12 (DMEM/F12) was supplemented with GlutaMAX (Thermo Fisher, Waltham, MA, USA) and with 10% fetal bovine serum (FBS, Dutscher, Brumath, France) and maintained at 37 °C in an atmosphere containing 5% CO_2_. Cells were routinely tested for mycoplasma contamination using the MycoAlertTM (Lonza) test and passaged twice per week before they reached 80–85% confluency. The ARPE-19 cells were seeded at a density of 7500 cells/cm^2^ into 75 cm^2^ flasks (Sarstedt, Marnay, France) for the immunoblotting experiments, 12-well plates (Sarstedt, Marnay, France) for flow cytometry, 24-well plates (Sarstedt, Marnay, France) for immunofluorescence, or 48-well plates (Sarstedt, Marnay, France) for the ELISA and DCFDA assays, in DMEM/F12 supplemented with 10% FBS. After 72 h of cell culture, nonpolarized ARPE-19 cells (60–70% confluence) were washed twice with Hank’s balanced salt solution (HBSS, Dutscher, Brumath, France) and then incubated in DMEM/F12 supplemented with 1% of FBS for 24 h. Cells were further incubated with an increasing concentration of RWE (30, 50, and 100 µg/mL) or RSV (20 µM) or with the solvent alone (0.1% of ethanol 70% *v*/*v*). After 24 h of pretreatment, cells were further treated with rVEGF, H_2_O_2_, AAPH, or LPS + IFN-γ at the indicated concentrations and times of treatments. In any of the experimental conditions, control cells were treated with the corresponding solvents (used for compound solubilization) alone and did not exceed 0.1% for ethanol 70%, 0.02% for DMSO, and 0.2% for H_2_O.

#### 2.3.2. PBMC-Derived Macrophages

Buffy coats of fresh blood from four healthy male donors with different blood types (B+, A−, O+, and AB+) were obtained from the Etablissement Français du Sang (EFS). In order to separate peripheral blood mononuclear cells (PBMC), buffy coats were diluted with phosphate-buffered saline (PBS, Dutscher, Brumath, France) 1× (1:1, *v*/*v*) and loaded on the top of a Ficoll (CMSMCLO1, Eurobio, Les Ulis, France). After 20 min of centrifugation at 700× *g* (without deceleration), PBMC were collected and washed twice with PBS 1×. PBMC were then seeded at a density of 800,000 cells/cm^2^ into 48-well plates in DMEM/F12 without FBS. After 2 h, the cells were washed twice, and adherent monocytes were cultivated in DMEM/F12 10% FBS with 100 ng/mL GM-CSF (130-093-866, Miltenyi Biotec, Bergisch Gladbach, Germany). After 6 days of culture (medium was changed every 2 days), the cells were further treated with the solvent (0.1% ethanol 70%) alone as a control or with increasing concentrations of red wine extract (RWE, 30, 50, and 100 µg/mL) or resveratrol (RSV, 20 µM). After 24 h, cells were activated with LPS O55:B5 (10 ng/mL) and IFN-γ (25 ng/mL) or water as the control. After 4 h of LPS + IFN-γ stimulation, cells were again challenged with increasing concentrations of RWE, RSV, or solvent alone, as described above, for an additional 20 h.

### 2.4. ELISA

Cells (ARPE-19 or PBMC) were seeded and treated in 48-well plates, as previously described. At the end of the treatments, cell culture supernatants were collected, and the secretion levels of human VEGF-A (446504, BioLegend, Amsterdam, the Netherlands), IL-6 (430504, BioLegend, Amsterdam, the Netherlands), and IL-8 (431504, BioLegend, Amsterdam, the Netherlands) were measured by ELISA according to the manufacturer’s protocols. Briefly, 96-well plates were coated with anti-VEGF-A, anti-IL-6, or anti-IL-8 capture antibodies overnight (O/N) at 4 °C. After washing, the standards provided in the kits were prepared in the assay diluent according to the manufacturer’s protocols. Cell supernatants of each experimental condition were diluted with a sample diluent, loaded onto a 96-wells plate, and incubated for 2 h at room temperature (RT) on a microplate shaker. At the end of incubation, individual wells were washed twice, and then, 100 µL of anti-VEGF-A, anti-IL-6, or anti-IL-8 detection antibodies were added and incubated at RT for 1 h on a microplate shaker. After washing, 100 µL of avidin conjugated to horseradish peroxidase (HRP) were loaded onto the plate and incubated for 30 min at RT in the dark; then, 100 µL of substrate were added and incubated for an additional 10 min. The reaction was stopped by adding 100 µL of stop solution (H_2_SO_4_, 1N), and then, the absorbance was measured at 450 nm using Perkin Elmer Multimode Plate Reader Envision. To calculate the cytokine concentrations, the standard curves for each assay were plotted with GraphPad Prism 8.3.0 software (GraphPad Software, La Jolla, San Diego, CA, USA) using the 4-parameter sigmodal curve fit.

### 2.5. Western Blot Analysis

ARPE-19 cells were seeded and treated in 75 cm^2^ flasks (562,500 cells/flask). Cells were collected; washed twice with cold PBS 1×; centrifuged (5 min, 450× *g*, 4 °C); and then lysed 30 min on ice with radioimmunopecipitation assay (RIPA) buffer (50 mM Tris-HCl, 150 mM sodium chloride, 0.1% sodium dodecyl sulfate, 0.5% sodium deoxycholate, 1% NP40, and pH8) supplemented with a phosphatase inhibitor, sodium fluoride (50 mM), protease inhibitor phenylmethylsulfonyl fluoride (PMSF) (100 µM, Sigma-Aldrich, St. Quentin Fallavier, France), and protease inhibitor cocktail (Roche, Boulogne-Billancourt, France). After centrifugation (20 min, 16,000× *g*, 4 °C), cell debris was eliminated, and protein quantification was performed using the QuantiPro^TM^ BCA assay kit (QPBCA, Sigma-Aldrich, St. Quentin Fallavier, France) and by using bovine serum albumin (BSA) as a standard. Samples containing 25–60 µg of proteins were prepared in Laemmli loading buffer (60 mM Tris HCl, 10% glycerol, 2% sodium dodecyl sulfate, pH6.8, 100 mM dithiothreitol, and 0.002% bromophenol blue) and were then heated for 5 min at 95 °C. Proteins were resolved by 7.5–12.5% Acrylamide TGX Stain-Free™ FastCast™ (Bio-Rad, 1610185, Marnes-la-Coquette, France) and then transferred onto 0.2 µm nitrocellulose membrane Trans-Blot Turbo (Bio-Rad, 1704271, Marnes-la-Coquette, France). Nonspecific binding sites were blocked using either 5% of bovine serum albumin (BSA) or 5% of skimmed milk in Tris-buffered saline (TBS)–Tween 20 0.1% for 1 h at RT before O/N incubation at 4 °C with specific primary antibodies. Primary antibodies were then detected with appropriate HRP-conjugated secondary antibodies (111-035-144 and 115-035-146, Jackson ImmunoResearch, Interchim, Montlucon, France) for 1 h at RT, followed by exposure to enhanced chemiluminescence (ECL, 1705032, Bio-Rad, Marnes-la-Coquette, France). The signal was acquired with the ChemiDoc^TM^ XRS imaging system (Bio-Rad, Marnes-la-Coquette, France). The densitometry of the blot was analyzed with Image Lab^TM^ version 6.0.1 software (Bio-Rad), and HSC70 was used as the internal loading control.

### 2.6. Cell Viability Assays

Cell viability was determined by crystal violet staining (Sigma-Aldrich, St. Quentin Fallavier, France). After treatments, cells were washed twice with PBS 1× and then stained with crystal violet solution (2.3% crystal violet, 0.1% ammonium oxalate, and 20% ethyl alcohol, #HT90132, Sigma-Aldrich, St. Quentin Fallavier, France) for 15 min at RT and then rinsed with water. After drying, crystal violet stain was dissolved in acetic acid 33%, and then, the absorbance was read at 590 nm using a PerkinElmer^®^ Multimode Plate Reader, Envision.

### 2.7. Intercellular Reactive Oxygen Species (ROS) Measurement

ARPE-19 cells were seeded at a density of 7500 cells/cm^2^ in 48-well plates in DMEM/F12 supplemented with 10% FBS. After 72 h of cell culture, the culture medium was removed, and cells were washed twice with Hank’s balanced salt solution (HBSS, Dutscher, Brumath, France) and then incubated in DMEM/F12 supplemented with 1% of FBS for 24 h. Cells were further incubated with ethanol 70% (vehicle control) and increasing concentrations of RWE (30, 50, 100 µg/mL) or RSV (20 µM). After 24 h of treatment, the cells were washed twice with HBSS and incubated with 10 µM of 2′,7′-dichlorofluorescin diacetate (DCFH-DA, D6863, Sigma-Aldrich, St. Quentin, France) or with the solvent alone (DMSO, 0.02%) as a control for 30 min at 37 °C. After washing, ARPE-19 cells were treated with H_2_O_2_ or AAPH at the indicated concentrations or with the culture medium or H_2_O as the control.

At the end of the treatments, the fluorescence intensity (excitation = 488 nm; emission = 528 nm) was measured using PerkinElmer Multimode Plate Reader Envision, and the cell viability was assessed by crystal violet staining.

### 2.8. Immunofluorescence

ARPE-19 cells (7500 cells/cm^2^) were cultured in 24-well plates on coverslips. After treatment, cells were washed twice with PBS 1× and then fixed and permeabilized for 10 min on ice with glacial methanol. After washing cells twice with Tris-buffered saline (TBS) 1×, nonspecific sites were blocked for 1 h at RT with a solution containing 1% BSA and 0.2% Triton 100×. Next, cells were incubated O/N at 4 °C with anti-*p*-histone H2A.X (Ser139) primary antibody (#80312, Cell Signaling Technology, Ozyme, Saint-Cyr-l’Ecole, France) diluted at 1:200 in TBS 1× containing 1% BSA and 0.2% Triton 100×. After washing with TBS 1× containing 0.2% Triton 100×, the cells were incubated with secondary Alexa Fluor goat anti-mouse 488 (SAB4600387, Sigma-Aldrich, St. Quentin Fallavier, France) diluted at 1:500 in TBS 1× containing 1% BSA and 0.2% Triton 100×. After washing with TBS 1× containing 0.2% Triton 100×, coverslips were mounted in Prolong^®^ Gold Antifade with DAPI (P36941, Invitrogen, Thermo Fisher, Waltham, MA, USA) in the dark for 24 h. Images were acquired with AxioImager M2 (Zeiss) and analyzed with Zen 3.6 (blue edition, Zeiss, Rueil Malmaison, France) and Icy (v2.4, Institut Pasteur and France-BioImaging, Paris, Montpellier, France) software.

### 2.9. Flow Cytometry

ARPE-19 cells were seeded and treated in 12-well plates as previously described. After treatments, cells were collected and viable cells were stained with fixable viability stain 520 (564407, Becton Dickinson, Franklin Lakes, NJ, USA) for 15 min at RT in the dark. After washing, cells were permeabilized with glacial methanol for 10 min on ice. Nonspecific sites were blocked for 10 min at RT with a saturation buffer (PBS 1×, 1% BSA, and 0.2% Triton 100×), and then, cells were incubated with Alexa Fluor^®^ 647 anti-*p*-histone H2A.X (Ser139) antibody (613408, BioLegend, San Diego, CA, USA) in the saturation buffer (1:100) for 30 min at RT. After washing, an analysis was performed using an Aurora cytometer (Cytek^®^ Biosciences, Fremont, CA, USA) with Spectroflo^®^ software (Biosciences, Torrance, CA, USA), and the data were analyzed using FlowJo v10 software (version 10, Tree Star, Ashland, OR, USA).

### 2.10. Statistics

Data were expressed as the means ± standard error of the mean (SEM) for at least three independent experiments (2–4 biological replicates per independent experiment). Statistical analyses were carried out with GraphPad Prism 8.3.0 software (GraphPad Software, La Jolla, San Diego, CA, USA). Data were compared among experimental groups using one-way ANOVA followed by Tukey’s multiple comparison test, two-way ANOVA followed by Dunnett’s multiple comparison test, or the Kruskal–Wallis test with Dunn’s post hoc analysis as appropriate after having checked the data for normal distribution (Shapiro–Wilk test) and variance homogeneity. All *p*-values were two-tailed, and *p*-values less than 0.05 were considered significant (* *p* < 0.05, ** *p* < 0.01, *** *p* < 0.001, and **** *p* < 0.0001).

## 3. Results

### 3.1. RWE Prevents VEGF-A Production Induced by rVEGF and H_2_O_2_ in ARPE-19 Cells

We have previously demonstrated that the contents of bioactive substances in wine and, more specifically, polyphenols could influence the biological effects observed in various biological systems. These qualitative and quantitative variations mainly result from the conditions of vinification but, also, from the grape variety and from exogenous factors (biotic or abiotic stress) [29,32,33]. In order to compare the activity of our red wine dry extract to a standard which properties are now well established, RSV, we determined the quality and quantity of the phenolic compounds contained in the extract by HPLC, as previously described [30]. We can observe in Table 1 the different polyphenols characterized in the dry extract and their respective concentrations. This table shows a high proportion of phenolic acids and flava-3-ols compared to the amounts of anthocyanins, flavonols, and stilbenes (Table 1).

**Figure 1:**
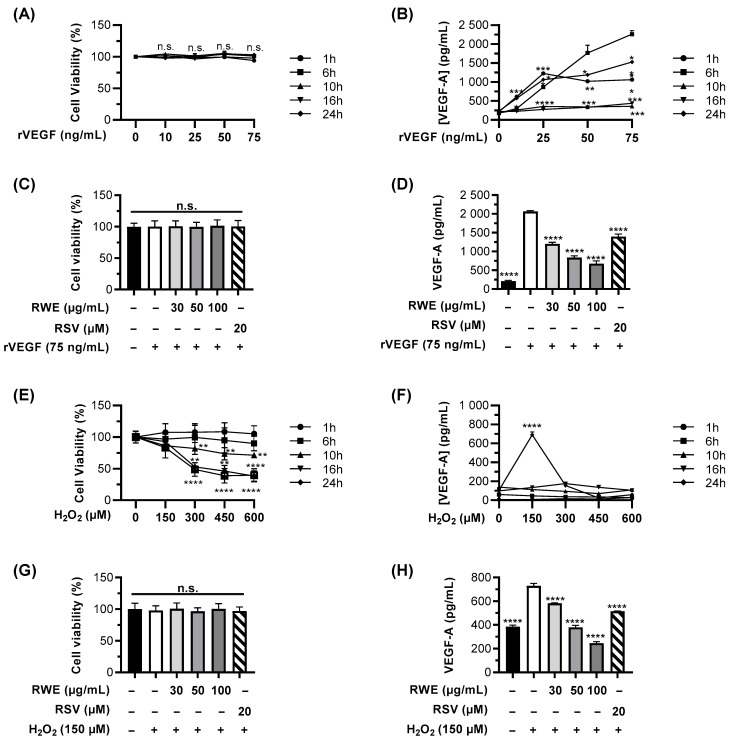
RWE prevents both rVEGF and H_2_O_2_-induced VEGF-A secretion in ARPE-19 cells. (**A**,**B**) ARPE-19 cells were treated for 1, 6, 10, 16, and 24 h with increasing concentrations of human recombinant VEGF (rVEGF) (0, 10 25, 50, and 75 ng/mL) or with the vehicle alone (0.1% H_2_O). After treatments, the cells’ viability was assessed by crystal violet staining (**A**), and the VEGF-A-secreted levels were measured in cell supernatants by ELISA (**B**). (**C**,**D**) ARPE-19 cells were treated for 24 h with increasing concentrations of RWE (30, 50, and 100 µM); RSV (20 µM); or with the vehicle alone (0.1% ethanol 70%) and then treated for 6 h with 75 ng/mL rVEGF or with the vehicle alone (0.1% H_2_O). After treatments, the cells’ viability was assessed by crystal violet staining (**C**), and the VEGF-A-secreted levels were measured in cell supernatants by ELISA (**D**). (**E**,**F**) Cells were treated for 1, 6, 10, 16, and 24 h with increasing concentrations of H_2_O_2_ (0, 150, 300, 450, and 600 µM) or with the vehicle alone. After treatments, the cells’ viability was assessed by crystal violet staining (**E**), and the VEGF-A-secreted levels were measured in cell supernatants by ELISA (**F**). (**G**,**H**) Cells were treated for 24 h with increasing concentrations of RWE (30, 50, and 100 µM); RSV (20 µM); or with the vehicle alone (0.1% ethanol 70%) and then treated for 24 h with 150 µM H_2_O_2_ or with the vehicle alone. After treatments, the cells’ viability was assessed by crystal violet staining (**G**), and the VEGF-A-secreted levels were measured in cell supernatants by ELISA (**H**). For the viability assays, data are expressed as a mean percentage relative to the control cells (treated with vehicles alone) ± SEM of three independent experiments (4 biological replicates per independent experiment). For kinetics (**A**,**B**,**E**,**F**), the *p*-values were determined by a two-way ANOVA followed by Dunnett’s multiple comparison test and compared to the 6 h timepoint. (**C**,**G**) The *p*-values were determined by a one-way ANOVA followed by Tukey’s multiple comparison test. The *p*-values ≤ 0.05 were considered significant (n.s. nonsignificant). For the ELISA assays (**D**,**H**), the data are expressed as a mean in pg/mL ± SEM of 3–5 independent experiments (2–4 biological replicates per independent experiment). The *p*-values were determined by a one-way ANOVA followed by Tukey’s multiple comparison test. The *p*-values ≤ 0.05 were considered significant (* *p* < 0.05, ** *p* < 0.01, *** *p* < 0.001, and **** *p* < 0.0001 comparing data to rVEGF- or H*_2_*O*_2_*-treated cells).

In order to determine whether RWE could affect the different molecular steps leading the development and progression of AMD, we first evaluated the ability of RWE to prevent the secretion of VEGF-A contributing to neoangiogenesis, which is the final step of the AMD process. We previously showed that this extract was able to decrease the basal level of VEGF-A in ARPE19 cells. However, what is the effect of RWE when its production is induced by exogenous factors? First, we evaluated the toxicity of increasing concentrations of recombinant human VEGF-165 (rVEGF) protein (10, 25, 50, and 75 ng/mL) after 1, 6, 10, 16, and 24 h of treatment. As shown in Figure 1A, none of the concentrations and time of treatment used had an impact on the cell viability, which was assessed by crystal violet staining. In the same experimental conditions, we further measured the secretion levels of VEGF-A in the cell supernatants. As shown in Figure 1B, rVEGF induced a maximal VEGF-A secretion by ARPE-19 cells after 6 h of treatment when used at 75 ng/mL. Based on this observation, we next evaluated the ability of RWE to counteract the increase in VEGF-A induced by 75 ng/mL of rVEGF after an optimal stimulation of 6 h. Very interestingly, 24 h of pretreatment with RWE was able to significantly decrease VEGF-A secretion in a concentration-dependent manner in ARPE-19 cells. Indeed, rVEGF-induced VEGF-A secretion was reduced by 42%, by 59%, and by 67% when cells were, respectively, pretreated with 30, 50, and 100 µg/mL RWE (Figure 1D) without impacting the cell viability (Figure 1C). Although RSV decreased VEGF-A secretion significantly (32% with 20 µM of RSV compared to the control), the percentage of inhibition was clearly lower compared to the RWE and whatever the concentration tested. Extracellular VEGF-A is not the only inducer able to stimulate the secretion of VEGF-A by RPE cells. Indeed, the molecules responsible for oxidative stress such as hydrogen peroxide (H_2_O_2_) are also at the origin of the increase in VEGF-A. To evaluate the ability of RWE to counteract oxidative stress-induced VEGF-A secretion by ARPE-19 cells, we first determined the optimal concentrations and time of treatment with H_2_O_2_. We thus treated the cells with increasing concentrations of H_2_O_2_ (150, 300, 450, and 600 µM) for 1, 6, 10, 16, and 24 h and determined the cell viability (Figure 1E) and, in cell supernatants, the VEGF-A levels by ELISA (Figure 1F). Only 150 µM H_2_O_2_ was able to significantly increase VEGF-A secretion as compared to the control cells after 24 h of treatment without impacting the ARPE-19 cell viability (Figure 1E,F). Next, cells were treated for 24 h with increasing concentrations of RWE or RSV, as previously described, and were then treated with 150 µM H_2_O_2_ for an additional 24 h. In these experimental conditions, no significant toxicity was observed with any of the treatments (Figure 1G). Nevertheless, the data showed that VEGF-A secretion induced by 150 µM H_2_O_2_ was significantly reduced by the RWE pretreatment in a concentration-dependent manner, with 100 µg/mL RWE being the most efficient (reduction of VEGF-A secreted levels by 67% compared to H_2_O_2_ alone) (Figure 1H). Regarding RSV, it reduced, to a similar extent, the H_2_O_2_-induced VEGF-A levels (29% vs. 20% for RSV and 30 µg/mL RWE, respectively) but was less efficient than RWE at 50 and 100 µg/mL.

### 3.2. RWE Inhibits ROS Levels Induced by Oxidative Stress in ARPE-19 Cells

**Figure 2:**
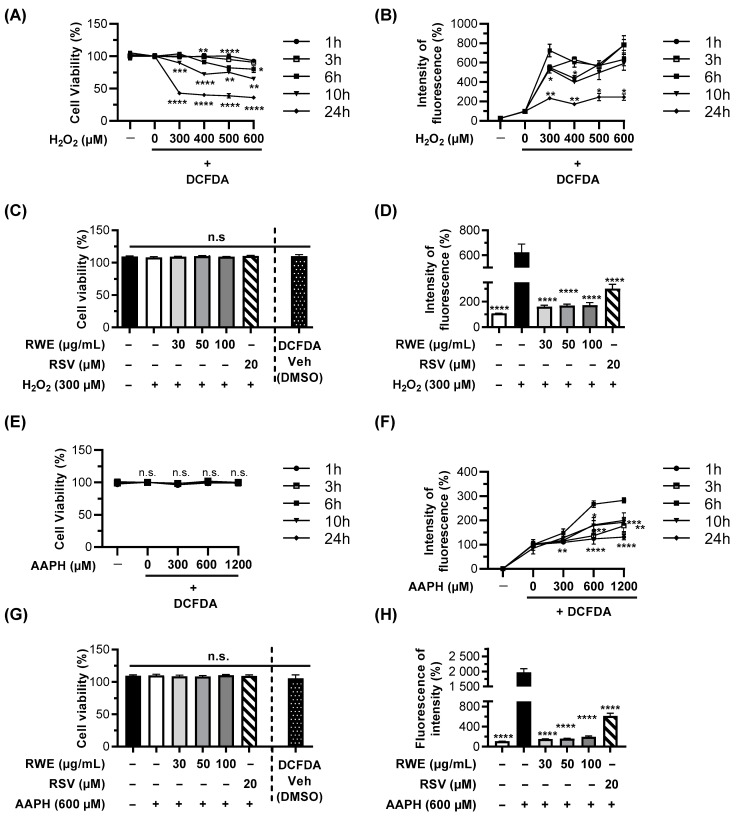
RWE prevents H_2_O_2_- and AAPH-induced oxidative stress in ARPE-19 cells. (**A**,**B**) ARPE-19 cells were incubated for 30 min with the ROS-sensitive probe DCFDA (10 µM) or with the vehicle alone (0.02% DMSO). Then, cells were treated for 1, 6, 10, 16, and 24 h with increasing concentrations of H_2_O_2_ (0, 300, 400, 500, and 600 µM) or with the vehicles alone (0.2% culture medium; -DCFDA: −). After treatments, the cells’ viability was assessed by crystal violet staining (**A**), and the ROS levels were quantified by flow cytometry (**B**). (**C**,**D**) Cells were treated 24 h with increasing concentrations of RWE (30, 50, and 100 µg/mL); RSV (20 µM); or with the vehicle alone (0.1% ethanol 70%). After 24 h, the cells were incubated with 10 µM DCFDA or the vehicle alone (0.02% DMSO) for 30 min, followed by 6 h of treatment with H_2_O_2_ (300 µM). Thereafter, the cells’ viability and ROS levels were determined as in (**A**,**B**). (**E**,**F**) ARPE-19 cells were incubated for 30 min with the ROS-sensitive probe DCFDA (10 µM) or with the vehicle alone (0.02% DMSO). Then, the cells were treated for 1, 6, 10, 16, and 24 h with increasing concentrations of AAPH (0, 300, 600, 1200, and 2400 µM) or with the vehicles alone (0.2% H_2_O; -DCFDA: −). After treatments, the cells’ viability was assessed by crystal violet staining (**E**), and the ROS levels were quantified by flow cytometry (**F**). (**G**,**H**) Cells were treated 24 h with increasing concentrations of RWE (30, 50, and 100 µg/mL); RSV (20 µM); or with the vehicle alone (0.1% ethanol 70%). After 24 h, the cells were incubated with 10 µM DCFDA or the vehicle alone (0.02% DMSO) for 30 min, followed by 6 h of treatment with AAPH (600 µM). After treatments, the cells’ viability was assessed by crystal violet staining (**G**), and the ROS levels were quantified by flow cytometry (**H**). For the kinetics in (**A**,**B**,**E**,**F**), the *p*-values were determined by a two-way ANOVA followed by Dunnett’s multiple comparison test and compared to the 6 h timepoint. (**C**,**G**) The *p*-values were determined by a one-way ANOVA followed by Tukey’s multiple comparison test. The *p*-values ≤ 0.05 were considered significant (n.s. nonsignificant). (**D**,**H**) The data are expressed as the mean DCFDA fluorescence intensity ± SEM of 3–5 independent experiments (2–4 biological replicates per independent experiment). The *p*-values were determined by a one-way ANOVA followed by Tukey’s multiple comparison test. The *p*-values ≤ 0.05 were considered significant (* *p* < 0.05, ** *p* < 0.01, *** *p* < 0.001, and **** *p* < 0.0001 comparing the data to H_2_O_2_- or AAPH-treated cells).

Therefore, H_2_O_2_ can contribute to the progression of the later processes of the disease through the production of VEGF-A. Furthermore, oxidative stress has been described as crucial in the early events of AMD with, in particular, the production of ROS, which induces several harmful effects. In order to show the ability of RWE to disrupt the production of ROS in human RPE cells, we first sought to determine the optimal conditions for oxidative stress-induced ROS production in the ARPE-19 cell line. Thus, we achieved kinetics from 1 to 24 h by stimulating ARPE-19 RPE cells with two well-known oxidative stress inducers, H_2_O_2_ and 2,2′-azobis(2-methylpropionamidine) dihydrochloride (AAPH) (Figure 2A,B,E,F). The cells were treated with increasing concentrations of H_2_O_2_ and AAPH as indicated, and then, the cell viability was assessed by crystal violet staining and ROS production by flow cytometry using the ROS-sensitive probe DCFDA. As a control, the cells were treated with the vehicles alone (−). Compared to AAPH, which do not induce cytotoxicity in the ARPE-19 cell line, H_2_O_2_, when used at high concentrations and for long times of incubation (10 to 24 h), reduced the cell viability by around 50% (Figure 2A,E), which translated into lower H_2_O_2_-induced ROS production (Figure 2B). According to the ROS curves obtained by the DCFDA assays, we thus selected 300 µM for H_2_O_2_ and 600 μM for AAPH in the subsequent experiments, since they induced significant ROS production as compared to the control cells (increases by 7- and 17-fold, respectively) without affecting the cell viability. We found that the pretreatment of cells with RWE significantly and strongly reduced H_2_O_2_ and AAPH-induced ROS production from the lowest concentration, 30 µM, allowing ROS to return to the basal levels (Figure 2D,H). As expected, the effect of RWE on ROS production was not related to cytotoxicity (Figure 2C,G). In this case, RSV also induced a significant reduction in the H_2_O_2_ and AAPH-induced production of ROS by 49% and 69% respectively, but it was not able to return to the basal level, as seen with RWE. The oxidative stress induced by H_2_O_2_ was associated with increases in the protein expression of nuclear factor erythroid 2-related factor 2 (Nrf2), a key transcription factor that regulates gene expressions for antioxidant enzymes such as Heme-Oxygenase 1 (HO-1) [34] (Supplementary Figure S1). RWE, to a greater extent than RSV alone, tended to rescue H_2_O_2_-induced Nrf2 expression but not that of HO-1. This suggests that the protective effects of RWE against oxidative stress may occur through the activation of cellular antioxidant defenses (Supplementary Figure S1).

### 3.3. RWE Prevents Oxidative Stress-Induced DNA Damage in RPE Cells

**Figure 3:**
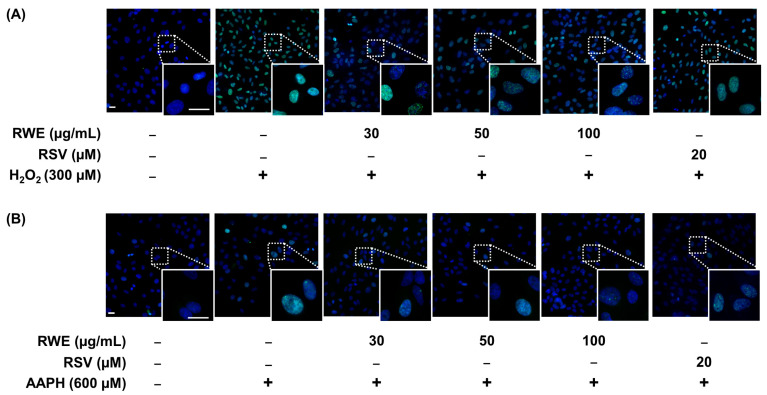
RWE prevents oxidative stress-induced DNA damage in ARPE-19 cells. Cells were treated with increasing concentrations of RWE (30, 50, and 100 µg/mL); RSV (20 µM); or with the vehicle alone (0.1% ethanol 70%). After 24 h, the cells were incubated with (**A**) H_2_O_2_ (300 µM) or with (**B**) AAPH (600 µM) for 6 h. γH2AX expression was analyzed by fluorescence microscopy (×20 and ×63 magnifications, scale bar = 20 µm). Nuclei (blue fluorescence) and γH2AX green fluorescence. Representative images of three independent experiments are shown.

In the first steps of AMD, oxidative stress can induce progressive retinal cellular damage, which leads to protein misfolding, functional anomalies, and DNA damage that trigger RPE cell dysfunctions. One of the most commonly used DNA oxidative stress markers is histone H2AX phosphorylation. Indeed, the phosphorylated histone H2AX (γH2AX), is usually used to assess DNA damage levels, being a marker of single- and double-stranded DNA breaks [35,36]. Firstly, we explored the ability of RWE to prevent DNA damage induced by oxidative stressors (i.e., H_2_O_2_ and AAPH) in ARPE-19 cells. Microscopic analyses highlighted the typical γH2AX foci after H_2_O_2_ treatment with 300 µM for 6 h, which was concomitant to ROS production (Figure 3A). Similarly, AAPH induced the same foci of γH2AX after 6 h of treatment with 600 µM of AAPH (Figure 3B).

**Figure 4:**
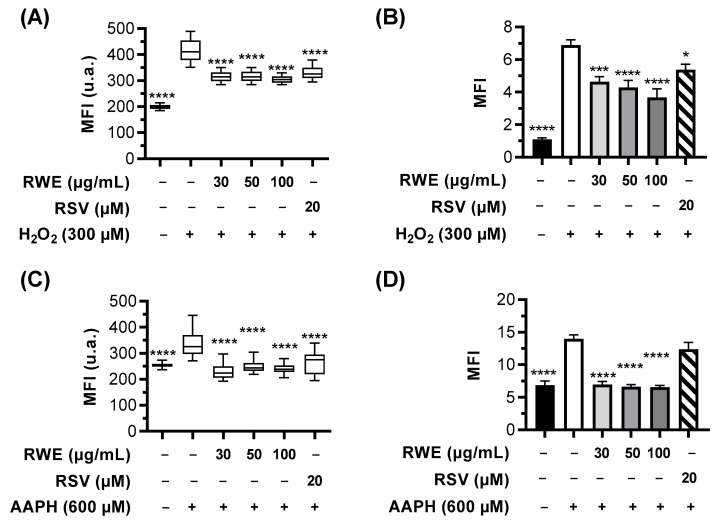
RWE prevents oxidative stress-induced DNA damage in ARPE-19 cells. (**A**,**B**) Cells were treated with increasing concentrations of RWE (30, 50, and 100 µg/mL); RSV (20 µM); or with the vehicle alone (0.1% ethanol 70%). After 24 h, the cells were incubated with H_2_O_2_ (300 µM) for 6 h. (**A**) Histograms of the mean fluorescence intensity (MFI) of γH2AX staining shown in Figure 3A. (**B**) Analysis of γH2AX staining in cells by flow cytometry. (**C**,**D**) Cells were treated with increasing concentrations of RWE (30, 50, and 100 µg/mL); RSV (20 µM); or with the vehicle alone (0.1% ethanol 70%). After 24 h, the cells were incubated with AAPH (600 µM) for 6 h. (**C**) Histograms of the mean fluorescence intensity (MFI) of γH2AX staining shown in Figure 3B. (**D**) Analysis of γH2AX staining in cells by flow cytometry. Fluorescence microscopy analyses were performed by measuring the MFI on merged pictures (n > 60 cells) with Icy software (v2.4) and were expressed as the mean MFI ± SEM of three independent experiments. The *p*-values were determined by Kruskal–Wallis followed by Dunn’s multiple comparison test and comparing the data to H_2_O_2_- or AAPH-treated cells. The *p*-values ≤ 0.05 were considered significant (**** *p* < 0.0001). Flow cytometry results were expressed as the mean MFI ± SEM (×10^4^) from 3 to 5 independent experiments with 2 to 4 biological replicates per experiment. The *p*-values were determined by a one-way ANOVA followed by Tukey’s multiple comparison test and comparing the data to H_2_O_2_- or AAPH-treated cells. The *p*-values ≤ 0.05 were considered significant (* *p* < 0.01, *** *p* < 0.001, and **** *p* < 0.0001).

Interestingly, pretreatment with three concentrations of RWE (30, 50, and 100 µM) for 24 h before oxidative stress induction prevented the appearance of green fluorescence, reflecting the increase in γH2AX foci (Figure 3A,B). A quantitative analysis of MFI measured with Icy software (v2.4) showed that H_2_O_2_ and AAPH induced significant increases in γH2AX foci by +108% and +37% compared to the control cells (cells treated with the vehicle alone). In addition, we observed that RWE pretreatment, whatever the concentration used, significantly prevented H_2_O_2_- and AAPH-induced rises in γH2AX foci by, respectively, around 25% and 30% (Figure 4A,C). In order to confirm the induction of DNA damage, we stained RPE cells with Alexa Fluor^®^ 647 anti-γH2A.X, and the MFI were measured by flow cytometry. These results confirmed the microscopic observations, showing that the treatment with 300 µM of H_2_O_2_ induces a strong increase in γH2AX staining compared to the control cells (by six-fold) (Figure 4B). The pretreatment with RWE significantly decreased H_2_O_2_-induced γH2AX in a concentration-dependent manner (33% for 30 µM, 38% for 50 µM, and 47% for 100 µM) compared to the H_2_O_2_ treatment (Figure 4B). RSV treatment with 20 µM had a modest inhibition of γH2AX (22%) compared to the RWE treatments. Similarly, AAPH induced a two-fold increase in the γH2AX level compared to the control cells, and the pretreatment with RWE decreased γH2AX even at the lowest concentration (30 µg/mL) and allowed a return to the basal levels (Figure 4D).

### 3.4. RWE Affects Activation of Key Regulators in the DNA Damage Response Pathway

DNA damage, whether single- or double-stranded breaks, results in activation of the DNA damage response (DDR) pathway. The two main pathways activated as a result of genomic damage are the ATM (ataxia telangiectasia-mutated)/Chk2 (checkpoint kinase 2) and RAD3-related (ATR)/Chk1 (checkpoint kinase 1) pathways. ATM and ATR proteins phosphorylate downstream histone H2AX (γH2AX), which then recruits other ATM and ATR substrates, notably Chk2 and Chk1 proteins. These proteins can then modulate the activity of key cell cycle regulators through the activation of transcription factor p53 [37,38]. Once activated, this triggers the G1/S, S, and G2/M checkpoints, thereby blocking cell cycle progression to enable either DNA repair or cell death induction. In order to investigate the effect of RWE on these DNA damage pathways, the expression of key proteins and their phosphorylation states were analyzed by Western blot in ARPE-19.

**Figure 5:**
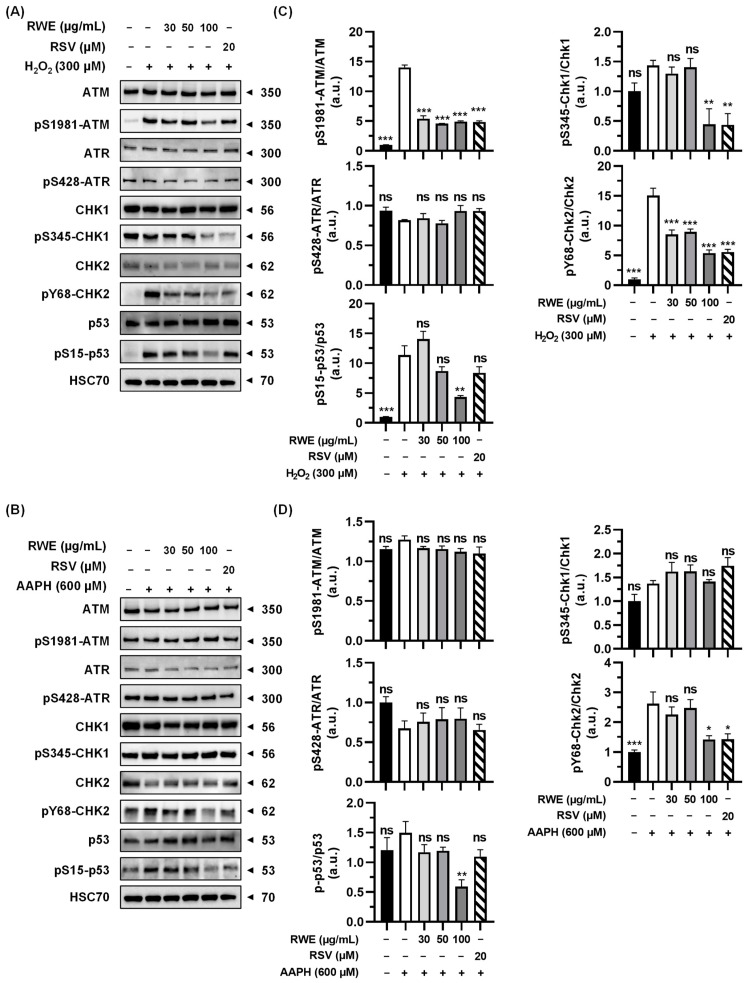
RWE affects H_2_O_2_- and AAPH-induced activation of key regulators of the DNA damage response (DDR) pathway in ARPE-19. Cells were treated with increasing concentrations of RWE (30, 50, and 100 µg/mL); RSV (20 µM); or the vehicle alone (0.1% ethanol 70%) for 24 h and were thereafter treated for 6 h with (**A**) H_2_O_2_ (300 µM) or by (**B**) AAPH (600 µM). Immunoblots of the key proteins involved in the DDR pathway are representative of three independent experiments. HSC70 was used as an internal loading control and was used for protein expression normalization. (**C**,**D**) Histograms corresponding to protein expression quantification. Data are the mean ratios between phosphorylated and total forms ± SEM (arbitrary units, u.a.) of 3 to 5 independent experiments. The *p*-value was determined by a one-way ANOVA followed by Tukey’s multiple comparison test comparing the data to H_2_O_2_- or AAPH-treated cells. *** *p*-value < 0.001, ** *p*-value < 0.01, * *p*-value < 0.05, and ns: nonsignificant.

First, we observed that H_2_O_2_ affects the ATM/Chk2 pathway. As shown in Figure 5A, H_2_O_2_ increases the phosphorylation of ATM and Chk2 by 14- and 15-fold, respectively, and a pretreatment with RWE is able to prevent these phosphorylations. Indeed, RWE prevents the phosphorylation of ATM even at the lowest concentration (around 65%) and, also, the phosphorylation of Chk2 in a dose-dependent manner (around 37% at 30 and 50 µg/mL and 62% at 100 µg/mL) (Figure 5C). Similarly, the pretreatment with RSV is also able to decrease the phosphorylation of ATM and Chk2 (around 60%). Similar results were observed with the phosphorylation of p53, with a significant increase of p53 phosphorylation induced by H_2_O_2_ by around 10-fold, and a pretreatment with RWE is able to reduce this phosphorylation at the strongest concentration (62% at 100 µg/mL). Interestingly, RSV is not able to significantly reduce the phosphorylation of p53. Then, we investigated the ATR/Chk1 pathway, and as shown in Figure 5A,C, H_2_O_2_ did not affect the expression and phosphorylation of ATR and Chk1. Nevertheless, a pretreatment with RWE at the strongest concentration (100 µg/mL) or RSV (20 µM) was able to significantly decrease the phosphorylation of Chk1 even below the basal control level.

In the same way, we studied the impact of AAPH treatment on the ATM/Chk2 and ATR/Chk1 pathways. Contrary to H_2_O_2_, AAPH does not impact the expression or the phosphorylation of ATM, ATR, Chk1, and p53 (Figure 5B). However, AAPH induces the phosphorylation of Chk2 2.5-fold, and the pretreatment with the highest concentration of RWE or with RSV is able to reduce this phosphorylation by around 45% (Figure 5B,D). Very interestingly, AAPH does not impact the expression or the phosphorylation of p53; however, the pretreatment with the highest concentration of RWE is able to reduce the phosphorylation of p53 by 56% compared to the control cells (Figure 5D).

### 3.5. RWE Prevents H_2_O_2_- and LPS/IFN-γ-Induced Inflammation in RPE Cells and Macrophages

It is well known that oxidative stress through ROS production and DNA damage contributes to inflammatory processes. Indeed, cigarette smoke concentrate induces ROS production and γH2AX nuclear foci in ARPE-19 cells, which are associated with the upregulation of proinflammatory cytokines IL-6 and IL-8 [39]. Indeed, RPE cells are involved in the regulation of the immune response in the retina, and dysfunctions in the RPE can lead to the secretion of proinflammatory cytokines such as IL-6 and IL-8, which maintain a proinflammatory environment [40]. These cytokines are crucial in AMD pathogenesis. Indeed, IL-6 has proangiogenic properties, the intraocular concentration of this cytokine being more closely correlated to the occurrence of macular edema than VEGF-A [41]. In this context, we have explored the RWE ability to prevent the secretion of IL-6 and IL-8 induced by oxidative stress (i.e., H_2_O_2_) in ARPE-19 cells.

**Figure 6:**
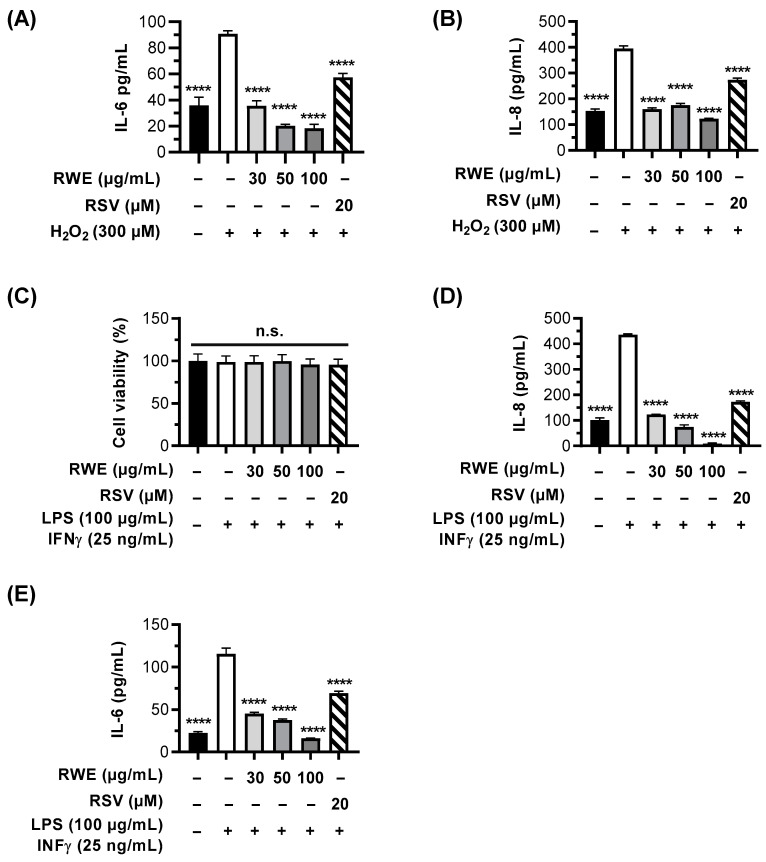
Anti-inflammatory effects of RWE on the secretion of proinflammatory cytokines IL-6 and IL-8 in ARPE-19 cells. Cells were treated with increasing concentrations of RWE (30, 50, and 100 µg/mL); RSV (20 µM); or the vehicle alone (0.1% ethanol 70%). After 24 h of treatment, the cells were incubated for 6 h with (**A**,**B**) H_2_O_2_ (300 µM) or with (**C**–**E**) LPS (100 µg/mL) and IFN-γ (25 ng/mL). The levels of (**A**,**D**) IL-6 and (**B**,**E**) IL-8 were measured by ELISA. Data were expressed as the mean in pg/mL ± SEM of 3–5 independent experiments (2–4 biological replicates per independent experiment). The *p*-values were determined by a one-way ANOVA followed by Tukey’s multiple comparison test comparing the data to H_2_O_2_- or LPS/IFN-γ-treated cells. The *p*-values ≤ 0.05 were considered significant (**** *p* < 0.0001). (**C**) Cell viability was measured by crystal violet staining. Data were expressed as the mean percentage relative to the control cells (treated with the vehicles alone) ± SEM of three independent experiments (4 biological replicates per independent experiment). The *p*-values were determined by a one-way ANOVA followed by Tukey’s multiple comparison test. The *p*-values ≤ 0.05 were considered significant (n.s. nonsignificant).

As expected, the treatment of cells with 300 µM of H_2_O_2_ for 6 h induced a strong secretion of IL-6 2.5-fold compared to the control. This latter was strongly reduced to the basal control levels by the pretreatment of cells with RWE 30 µg/mL and was even decreased to lower levels when using 50 and 100 µg/mL of RWE (reduction by 44% and 49%, respectively, compared to the control) (Figure 6A).

RSV is also usually described as an anti-inflammatory compound. In these oxidative stress conditions, polyphenol was able to decrease IL-6 secretion but at a lower level than RWE by 37% compared to H_2_O_2_ (Figure 6A). Similarly, IL-8 secretion was completely decreased with RWE pretreatment (Figure 6B). Indeed, ARPE-19 cells overproduce IL-8 under oxidative stress with H_2_O_2_ 2.5-fold compared to the control, which is completely returned to the basal level when RPE cells are pretreated with 30 or 50 µg/mL of RWE (Figure 6B). In view of these results, we then explored the capacity of RWE to counteract an overproduction of proinflammatory cytokines not by oxidative stress but by a common inducer of inflammation, such as a combination with lipopolysaccharide (LPS) and interferon gamma (IFN-γ). The quantification of IL-6 and IL-8 secretion by RPE cells revealed that LPS/IFN-γ increases IL-6 secretion 5-fold compared to the control (Figure 6D) and around 4.3-fold for IL-8 compared to the control (Figure 6E), without inducing any cytotoxicity (Figure 6C). The pretreatment of cells with RWE strongly decreases IL-6 production at the same basal level of the control with 50 and 100 µg/mL (Figure 6D). Very interestingly, RWE pretreatment decreases IL-8 secretion below the basal control level from 30 µg/mL and strongly decreases under the basal levels at 50 and 100 µg/mL (Figure 6E).

**Figure 7:**
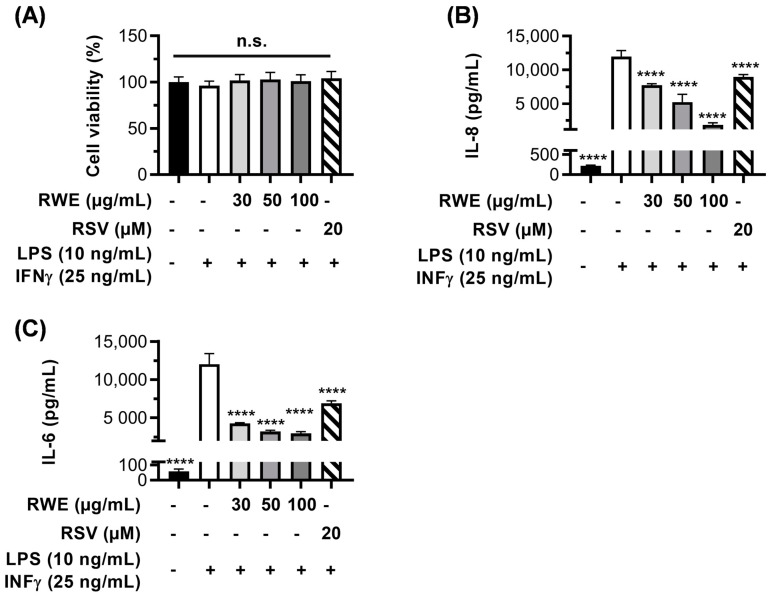
Anti-inflammatory effects of RWE on the secretion of proinflammatory cytokines IL-6 and IL-8 in human macrophages. After 6 days of culture, human PBMC were treated with increasing concentration of RWE (30, 50, and 100 µg/mL); RSV (20 µM); or the vehicle alone (0.1% ethanol 70%). After 24 h of treatment, the cells were incubated with LPS (10 ng/mL) and IFN-γ (25 ng/mL) to polarize cells into M1 macrophages or incubated with the vehicle alone. After 4 h of stimulation with LPS and IFN-γ, increasing concentrations of RWE (30, 50, and 100 µg/mL); RSV (20 µM); or the vehicle alone (0.1% ethanol 70%) were added into the medium for an additional 20 h. At the end of the treatments, the cells’ viability was assessed with crystal violet staining (**A**), and the supernatants were collected to measure by ELISA the secreted levels of (**B**) IL-6 and (**C**) IL-8. For the ELISA assays, the data were expressed as the mean in pg/mL ± SEM of 3–5 independent experiments (2–4 biological replicates per independent experiment). The *p*-value was determined by a one-way ANOVA followed by Tukey’s multiple comparison test. **** *p*-value < 0.0001 comparing the data to LPS/IFN-γ-treated cells. For cell viability, data were expressed as the mean percentage relative to the control cells (treated with the vehicles alone) ± SEM of three independent experiments (4 biological replicates per independent experiment). The *p*-values were determined by a one-way ANOVA followed by Tukey’s multiple comparison test. (n.s. nonsignificant).

These proinflammatory cytokines, which are produced in RPE cells under the effect of stress activators, are found in the aqueous humor of patients with AMD [42], and their levels have been correlated with neovascular retinal activity [43]. However, RPE cells are not the only immunosuppressive cells playing a role in the proinflammatory state seen in this disease. During AMD, monocytes from the blood circulation are recruited to the retina and choroid, where they differentiate into macrophages, especially into proinflammatory “M1”-type macrophages [15]. This type of macrophages is activated by multiple signals, including bacterial toxins such as LPS, and they secrete, in particular, proinflammatory cytokines such as IL-6 and IL-8 [44]. Considering the important role played by proinflammatory M1 macrophages in AMD progression, we next assessed whether the modulation of IL secretion by RWE in RPE cells could be similar in these immune cells. To this end, we isolated human peripheral blood mononuclear cells (PBMC) from four different male donors that we pre-differentiated with GM-CSF for 6 days. Thereafter, the cells were pretreated for 24 h with increasing concentrations of RWE (30, 50, and 100 µg/mL); RSV (20 µM); or with the vehicle alone before activating monocytes into M1 macrophages with LPS and IFN-γ. After 4 h of stimulation, the macrophages were treated for an additional 20 h with increasing concentrations of RWE or RSV, as previously described. At the end of the treatments, the supernatants were collected to measure the IL-6 and IL-8 secretion levels by ELISA (Figure 7B,C), and the cells were stained with crystal violet to assess the cell viability (Figure 7A). As expected, the treatment with LPS/IFN-γ induced IL-6 secretion that increased by 200-fold compared to the control with no significant toxicity (Figure 7A) and that was lowered in a dose-dependent manner with RWE (65%, 73%, and 76% at 30, 50, and 100 µg/mL, respectively) (Figure 7B). RSV decreased IL-6 secretion slowly compared to the different RWE concentrations, with 43% compared to LPS/IFN-γ (Figure 7B). Similarly, IL-8 secretion was increased by LPS/IFN-γ by around 50-fold compared to the control. Similar to IL-6, IL-8 secretion was inhibited in a dose-dependent manner by RWE, with 35%, 56%, and 84% at 30, 50, and 100 µg/mL, respectively (Figure 7C). As with ARPE-19, pretreatment with 20 µM of RSV decreased the production of the two proinflammatory cytokines compared to cells under stress conditions but at a lower level than with RWE treatment (43% for IL-6 and 25% for IL-8) (Figure 7B,C).

## 4. Discussion

Age-related macular degeneration (AMD) is the leading cause of central vision impairment in patients over the age of 65. This multifactorial disease is caused by both environmental and genetic risk factors [23]: smoking; air pollution; ultraviolet radiation; metabolic diseases (e.g., obesity, hypertension, and diabetes); consumption of dietary fats [24,25,26,27]; and genetic polymorphisms such as *cfh* and *arms2/hrta1* genes [28]. These factors can trigger AMD through the production of free radicals that create oxidative stress and inflammation in the retina. Both oxidative stress and inflammation are known for playing a critical role in the initiation and development of AMD [45]. Retinal pigment epithelial (RPE) cells are responsible for maintaining the retina homeostasis by providing nutritional and structural support; therefore, RPE are the primary target for AMD-associated oxidative stress [46]. RSV, a potent antioxidant compound, may be effective in reducing the risk of AMD in a same way that common antioxidants found in fruits and vegetables (vitamins C and E and carotenoids) have been shown to delay or prevent the development of ocular diseases [47].

Some studies have shown that food supplementation with resveratrol could have beneficial properties against eye disease [22], i.e., by reducing laser-induced choroidal neovascularization (CNV) in mice [48]. Furthermore, other polyphenols found in red wine (such as quercetin, epicatechin, and malvidin) have been reported to have potential positive effects in AMD by exerting antioxidant, anti-inflammatory, and antiangiogenic effects in both in vitro and in animal models of AMD [49,50,51,52,53]. In this context, we used a red wine extract (RWE) enriched with various properly identified and characterized polyphenols, and we describe, for the first time, the potential use of RWE to prevent early events, such as ROS production and proinflammatory IL secretion, in a human RPE cell line (ARPE-19).

It Is well established that polyphenols can both scavenge ROS and regulate signaling pathways involved in oxidative stress, inflammation, and angiogenesis. Indeed, the structure of polyphenol compounds is characterized by a poly hydroxyl group on an aromatic ring, which allows them to exhibit the radical scavenging of NO^·^ and OH^·^ [34]. In addition to their direct action on ROS, polyphenols also have biological functions by regulating the mitochondrial respiratory chain [54,55] and endogenous antioxidant enzymes such as superoxide dismutase, catalase, and glutathione peroxidase or various targets such as nuclear factor erythroid 2-related factor 2 (Nrf2) and the mammalian forkhead transcription factors of the O class (FOXOs) that are involved in the transcription of target antioxidant gene expression [34]. Among these polyphenols, the biological activities of RSV as an antioxidant and anti-inflammatory compound have been well described [56,57], particularly in the context of various ocular dysfunctions [22]. Furthermore, recent studies have shown that this hydroxystilbene is also able to act synergistically with other polyphenols to counteract many deleterious processes. It has now been clearly demonstrated that polyphenolic compounds that are said to have synergistic activities are highly effective against oxidation, inflammation, myocardial infarction, and a variety of other conditions [58,59,60,61]. In addition, natural polyphenols act synergistically with existing clinically approved drugs to improve anticancer activity [37,62,63,64]. We have addressed, in many pathological conditions, the potential use of a mixture from red wine that contains various polyphenols. We were thus able to reveal that the qualitative and quantitative compositions of polyphenols were decisive in the biological activity of these polyphenolic mixtures, because depending on the presence or absence of certain compounds, we observed synergistic effects, additives, or even opposing antagonistic effects [29]. Similarly, we were able to demonstrate, in a previous study on antitumor activity, that the relative concentrations of these compounds were also decisive and that it was the mixture from the dry red wine extract richest in polyphenols that exhibited the best antitumor activity in a model of chemo-induced carcinogenesis in rats [29]. Very interestingly, these extracts were able to alter tumor angiogenesis in many models through the reduction of VEGF-A [29,65,66]. This proangiogenic factor has a major importance in the progression of AMD and poor clinical prognosis. Se recently showed that the extract enriched in polyphenols was also able to reduce the secretion of VEGF-A in a retinal model of ARPE-19 cells by disturbing the VEGF-R2/mitogen-activated protein kinase (MAPK) signaling pathway [30]. This pathway is not the only one that leads to the overproduction of VEGF-A, and early AMD events can contribute significantly to VEGF-A production and, thus, to the triggering of angiogenesis, as observed during oxidative stress [39] or chronic inflammation [67,68].

Oxidative stress is the first step of AMD. It can lead to cell injury such as DNA damage, triggering RPE cell dysfunction. By inducing ROS production, nuclear damage appears in RPE cells, leading to phosphorylated-histone 2AX-immunoreactive (γH2AX) nuclear foci, which trigger an increase in the p53 protein [39]. The present study highlights that RWE was able to prevent H_2_O_2_- or AAPH-induced oxidative stress in human ARPE-19 RPE cells in a dose-dependent manner and, thus, protect RPE cells against DNA damage, as demonstrated by the decrease in γH2AX. Subsequently, this protective effect is associated with a downregulation of the DDR pathway involving the ATM/Chk2 and ATR/Chk1 proteins. Very interestingly, as observed in previous studies, RWE was more efficient than RSV alone in preventing oxidative stress and DNA damage, suggesting a potential synergy between polyphenol compounds at very low concentrations.

It is well known that oxidative stress resulting from ROS production and DNA damage contributes to inflammatory processes. Indeed, during aging or exposure to exogenous and endogenous factors, ROS production and γH2AX nuclear foci are induced in RPE cells in association with an upregulation of proinflammatory cytokines, particularly IL-6 and IL-8 [39]. Furthermore, drusen formed during the AMD process contain numerous proinflammatory interleukins and plasmatic proteins (such as C-reactive protein (CRP)) involved in acute inflammation [69]. Indeed, it has been shown that the plasma CRP levels are higher in patients with AMD. Similarly, high levels of IL-6 have been associated with the progression of AMD. Furthermore, many anatomopathological studies have demonstrated the presence of inflammatory cells from the early stage of AMD, notably preceding the appearance of retinal lesions. Particularly, mononuclear phagocytes are present at the edge of atrophic zones and are responsible for the degeneration of photoreceptors. Therefore, there is an amplification loop for inflammation, since there is chronic inflammation of the cells in the RPE that progressively alters their function and results in the formation of drusen. In our study, we show that RWE was able to prevent the secretion of proinflammatory cytokines (IL-6 and IL-8) in RPE cells induced by oxidative stress and by LPS, but RWE was also able to prevent these same cytokines in inflammatory cells such as macrophages. Interestingly, in these two cell types, RSV alone was able to prevent the secretion of IL-6 and IL-8, but the effect was not as marked as for RWE. Overall, RWE and RSV alone can be beneficial in the prevention of the events leading to AMD, but RWE appears to be more efficient.

Overall, these results, which were obtained using a polyphenolic cocktail on a human retinal cell line (ARPE-19) and human macrophages, pave the way for future studies. Our findings suggest that this formula may reduce or prevent events associated with AMD, thus slowing the progression of AMD and preventing the formation of new vessels. Nonetheless, as polyphenols are subjected to intensive metabolism by phase I/phase II enzymes [70,71,72], further studies are required to evaluate the ability of such a polyphenolic formulation to prevent AMD onset or progression and to analyze RWE metabolite profiles in target tissues using a mass spectrometry approach [48].

## 5. Conclusions

Our study demonstrated, for the first time, that a red wine extract (RWE) enriched in various polyphenols could counteract the early events of AMD, such as ROS production and proinflammatory IL secretion, in a human RPE cell line (ARPE-19). We showed that RWE can have an antiangiogenic effect and that it can prevent ROS and protect cells from DNA damage through the downregulation of key factors in the DNA damage response pathway, such as ATM, Chk2, and p53. These antioxidant effects are enhanced by anti-inflammatory benefits. Moreover, RWE and RSV are able to counteract the secretion of proinflammatory cytokines induced in ARPE-19 cells and human macrophages. These in vitro findings pave the way for further in vivo and clinical investigations on the health benefits of polyphenols and their metabolites.

## Figures and Tables

**Table 1:** Doses (µg/mL) of red wine extract (RWE) used in the study.

			Concentration (nM) of Compounds from Used Doses of RWE		
Compounds		mg/g of Extract	30 µg/mL	50 µg/mL	100 µg/mL
Phenolic Acids		51.25 × 10^−1^			
	Gallic Acid	24.07 × 10^−1^	424.49	707.49	1414.98
	Caftaric Acid	25.93 × 10^−1^	249.18	415.31	830.61
	Caffeic Acid	12.44 × 10^−2^	20.72	34.54	69.08
Flavan-3-ols		37.78 × 10^−1^			
	Catechin	90.98 × 10^−2^	94.04	156.74	313.47
	Epicatechin	33.30 × 10^−2^	34.42	57.36	114.73
	Procyanidin B1	16.72 × 10^−1^	84.40	140.66	281.33
	Procyanidin B2	49.92 × 10^−2^	25.20	41.99	83.98
	Procyanidin B3	18.76 × 10^−2^	9.47	15.78	31.57
	Procyanidin B4	17.52 × 10^−2^	8.84	14.74	29.48
Flavonols		2.35 × 10^−1^			
	Quercetin	19.62 × 10^−2^	19.48	32.47	64.94
	Quercetin 3-glucuronide	2.69 × 10^−2^	1.69	2.82	5.64
	Quercetin 3-rhamnoside	1.16 × 10^−2^	0.78	1.30	2.59
Stilbenes		5.61 × 10^−1^			
	cis-Resveratrol	15.70 × 10^−2^	20.64	34.39	68.79
	trans-Resveratrol	4.69 × 10^−2^	6.16	10.27	20.55
	cis-Piceid	4.57 × 10^−2^	3.52	5.86	11.73
	trans-Piceid	1.25 × 10^−2^	0.96	1.60	3.20
	trans-Piceatanol	1.53 × 10^−2^	1.88	3.13	6.27
	cis-epsilon-viniferin	1.21 × 10^−3^	0.08	0.13	0.27
	epsilon-viniferin	6.79 × 10^−3^	0.45	0.75	1.50
	omega-viniferin	3.57 × 10^−3^	0.24	0.39	0.79
	Pallidol	5.74 × 10^−2^	3.79	6.31	12.62
	Parthenocissin	3.48 × 10^−2^	2.30	3.83	7.65
	Isohopeaphenol	17.97 × 10^−2^	5.94	9.91	19.81
Anthocyanins		10.54 × 10^−1^			
	Delphindin 3-glucoside	14.48 × 10^−2^	8.68	14.46	28.92
	Cyanidin 3-glucoside	5.53 × 10^−3^	0.34	0.57	1.14
	Petunidin 3-glucoside	7.36 × 10^−2^	4.29	7.15	14.30
	Peonidin 3-glucoside	11.20 × 10^−2^	6.74	11.23	22.45
	Malvidin 3-glucoside	71.76 × 10^−2^	40.70	67.84	135.68

## Data Availability

The authors declare that all data supporting the findings of this study are available within the article and the Supplementary Materials.

## Supplementary Materials

The following supporting information can be downloaded at: https://www.mdpi.com/article/10.3390/cells12101408/s1, Figure S1: Effects of RWE on H_2_O_2_-induced protein expression of Nrf2 transcription factor and Heme-Oxygenase 1 (HO-1) in ARPE-19. Cells were treated with increasing concentrations of RWE (30, 50, 100 µg/mL), RSV (20 µM) or vehicle alone (0.1% ethanol 70%) for 24 h, and were thereafter treated for 6 h with H_2_O_2_ (300 µM). Immunoblots of Nrf2 and HO-1 are representative of three independent experiments. HSC70 was used as an internal loading control; Figure S2: Flow cytometry gating Strategies. Live cells Were stain withe Fixable viability staining (FVS 520). Gray histogram represents H2AX staining in ARPE-19 after treatment.
