# Supplementary material for: Red Wine Extract Prevents Oxidative Stress and Inflammation in ARPE-19 Retinal Cells
Source: Cells. 2023 May 17;12(10):1408. doi: 10.3390/cells12101408 (PMC10216569; doi:10.3390/cells12101408)
Supplement: Supplementary file 1 [file cells-12-01408-s001.zip › cells-2360491-supplementary.pptx]

## Slide 1
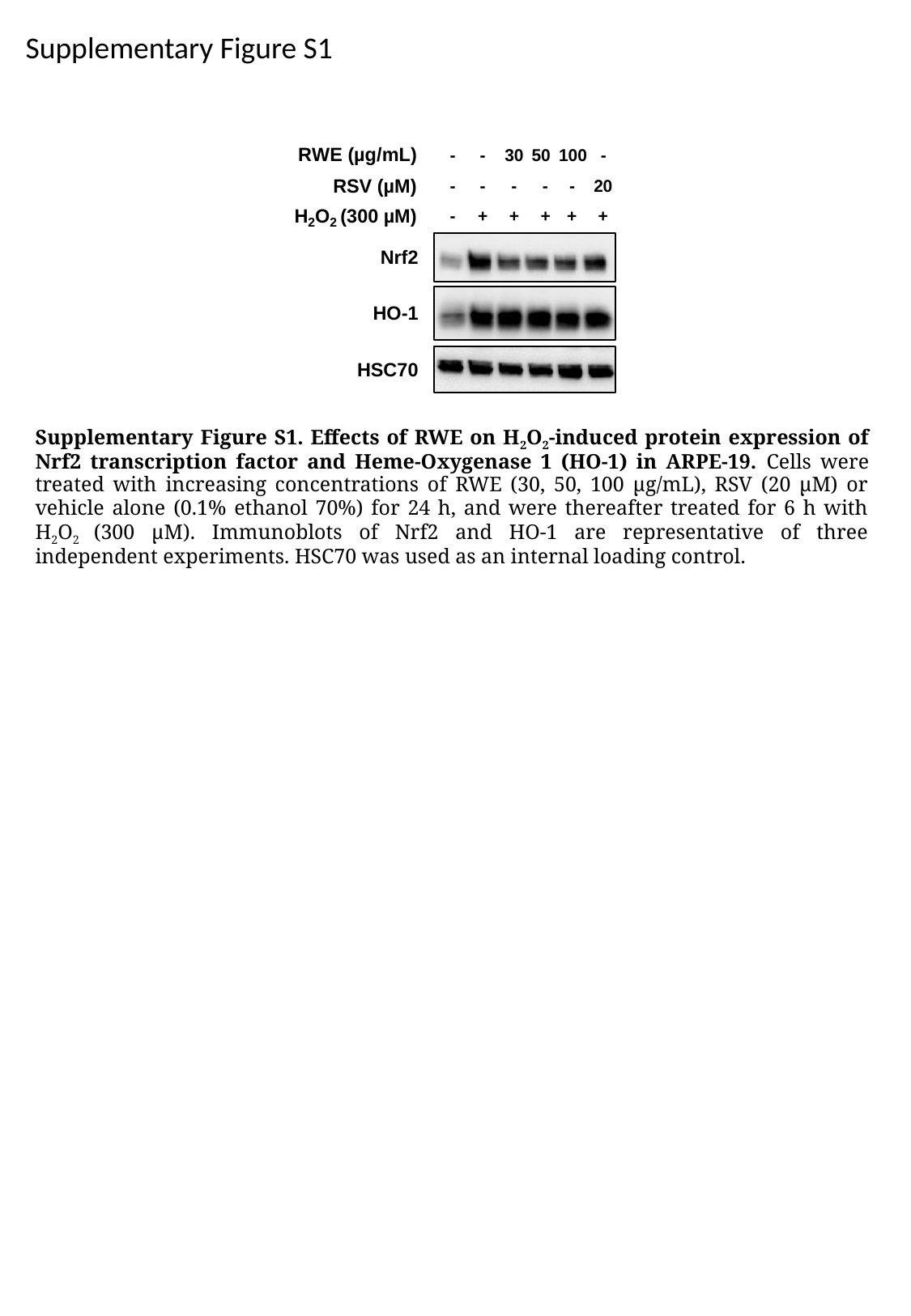

Supplementary Figure S1
Supplementary Figure S1. Effects of RWE on H2O2-induced protein expression of Nrf2 transcription factor and Heme-Oxygenase 1 (HO-1) in ARPE-19. Cells were treated with increasing concentrations of RWE (30, 50, 100 µg/mL), RSV (20 µM) or vehicle alone (0.1% ethanol 70%) for 24 h, and were thereafter treated for 6 h with H2O2 (300 µM). Immunoblots of Nrf2 and HO-1 are representative of three independent experiments. HSC70 was used as an internal loading control.

## Slide 2
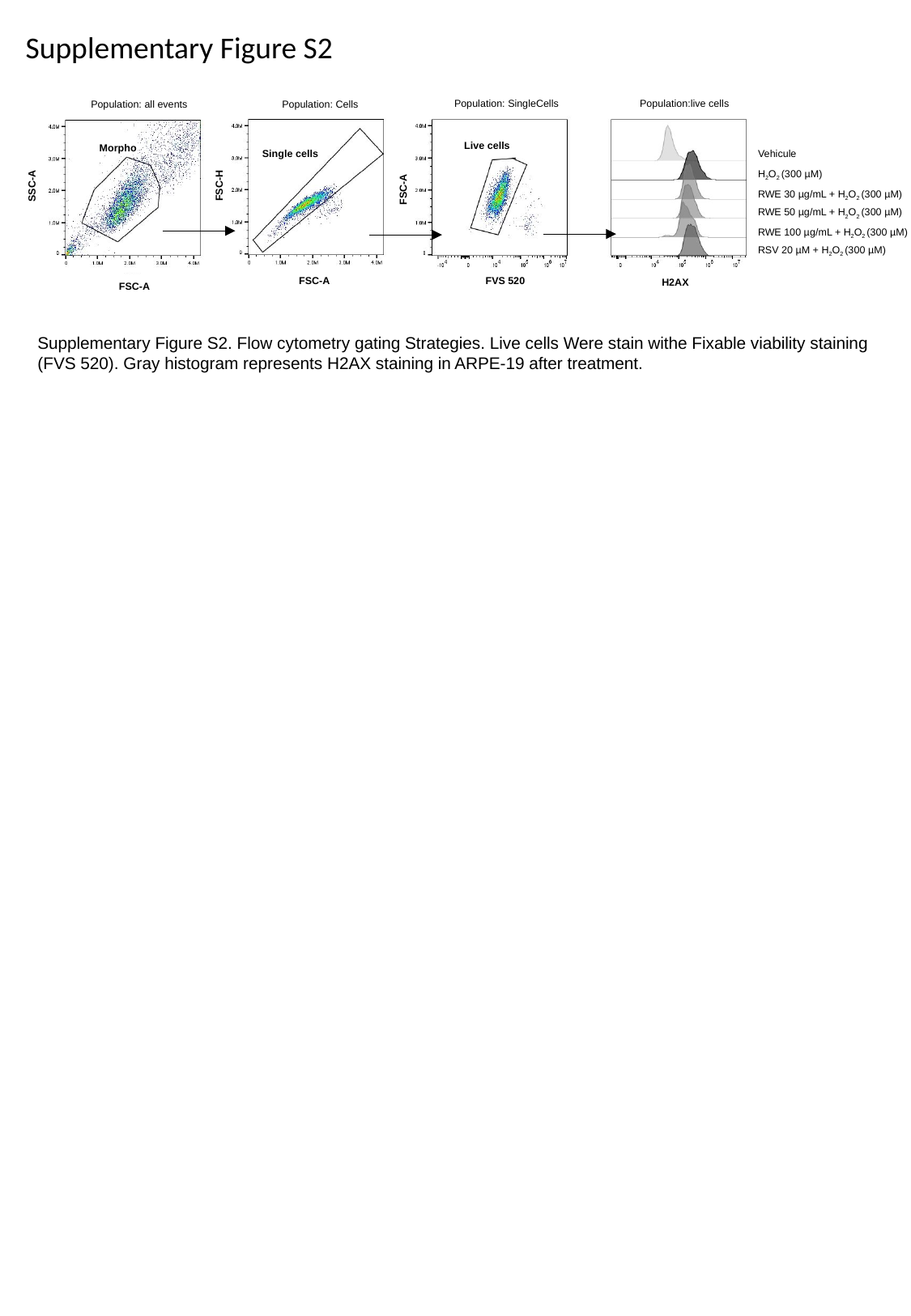

Supplementary Figure S2
Population:live cells
Population: SingleCells
Population: Cells
Population: all events
Live cells
Morpho
Single cells
Vehicule
H2O2 (300 µM)
FSC-H
SSC-A
FSC-A
RWE 30 µg/mL + H2O2 (300 µM)
RWE 50 µg/mL + H2O2 (300 µM)
RWE 100 µg/mL + H2O2 (300 µM)
RSV 20 µM + H2O2 (300 µM)
FSC-A
FVS 520
H2AX
FSC-A
Supplementary Figure S2. Flow cytometry gating Strategies. Live cells Were stain withe Fixable viability staining (FVS 520). Gray histogram represents H2AX staining in ARPE-19 after treatment.
